# Memristive property’s effects on the *I–V* characteristics of perovskite solar cells

**DOI:** 10.1038/s41598-017-05508-5

**Published:** 2017-07-20

**Authors:** Kai Yan, Bin Dong, Xinyu Xiao, Si Chen, Buxin Chen, Xue Gao, Hsienwei Hu, Wen Wen, Jingbo Zhou, Dechun Zou

**Affiliations:** 1 Beijing National Laboratory for Molecular Sciences, Key Laboratory of Polymer Chemistry and Physics of Ministry of Education, Center for Soft Matter Science and Engineering, College of Chemistry and Molecular Engineering, Peking University, Beijing, 100871 China; 2 Beijing Engineering Research Center for Active Matrix Display, Peking University, Beijing, 100871 China

## Abstract

The unfavorable *I–V* characteristics of perovskite solar cells (PSCs), such as the *I–V* hysteresis phenomena, have been one major obstacle for their future practical application. However, corresponding analysis based on traditional theories have shown non-negligible flaws and failed for satisfactory explanation. To present a novel mechanism, here we utilize for the first time the memristive property of the perovskite material to analyze the *I–V* characteristics of PSCs. The obtained joint physical model and the deduced equation may help solving the long-existent mysteries of the *I–V* characteristics of PSCs. On the basis of our analysis and memristor theory, we also propose an original device optimization strategy for PSCs, which may help further increase their performance to the limit.

## Introduction

Perovskite solar cells (PSCs) have been extensively investigated worldwide^1–4^. Given that the highest reported power conversion efficiency (PCE) of PSCs reached over 22%^5^ in just four years, let along their cost and manufacture advantages, PSCs have dwarfed their senior competitors, such as dye-sensitized solar cells (highest PCE 11.9%) and organic photovoltaic cells (highest PCE 11.5%); likewise, PSCs seem to be one step away in knocking down silicon solar cells^6^. To fulfil this goal, the “Gordian knot” here has to be cut first – those unfavorable yet widely observed *I–V* characteristics, such as the rich *I–V* hysteresis phenomena of PSCs – which affect device performances and hinder future practical applications^7^. Despite the urgency of this problem, satisfactory theory has yet to be proposed, while the reported ones have shown non-negligible drawbacks, such as the conflicting reports on ferroelectric property mechanism^8–10^, the time-scale discordance with the voltage scan rate in dynamic trapping/de-trapping processes of charge carriers^11, 12^, and the large time-scale variations among reported studies based on ion migration mechanism^13–15^. Besides, none single reported mechanism seems able to address all types of the *I*–*V* characteristics phenomena and some of the *I–V* characteristic phenomena have yet to be explained. Another troubling consequence of the lack of fundamental understanding on the *I–V* characteristics is the non-standardized test method for the *I–V* characteristics of PSCs. In fact, the device performance of PSCs can be manipulated by changing the test method. An example is shown in Figure S1 Supporting Information. In order to improve the reproducibility and transparence of the research results, *Nature Materials* released a checklist for photovoltaic research requiring authors to provide additional details on their testing methods^16^. However, to solve this problem once for all, we still have to determine the cause of the unique *I–V* characteristics of PSCs. Considering that traditional theories have failed for satisfactory explanation, maybe we should try some novel solutions.

Very recently, a few reported studies have successfully applied hybrid organic–inorganic perovskites to prepare memristors, which are next-generation non-volatile memory devices for information storage and processing; and the structure of these devices are similar to those of PSCs^17–19^. The electric characteristic of perovskite memristors is the non-overlapping *I–V* curves of forward and backward voltage scans, which is also similar to the basic feature of the *I–V* hysteresis phenomena of PSCs. The changeable resistance state of perovskite memristors is induced by the applied voltage, whose scope and scan rate are of the same values as those used in the *I–V* characteristic test of PSCs. In a reported work on perovskite memristors, both the memristive and photovoltaic properties of perovskite materials are utilized to fabricate a logical OR device^19^. It indicated that these two properties can co-exist in perovskite-based devices. Because of these obvious links between perovskite memristors and PSCs and the fact that memristor is actually one of the four fundamental circuit elements^20, 21^, the memristive property of perovskite may significantly influence the *I–V* characteristics of PSCs. However, this influence has been largely disregarded all this time.

Thus, we determined to investigate the possible effects of the memristive property of perovskite on the *I–V* characteristics of PSCs. And here we performed for the first time a quantified mechanism-level analysis of the effects of memristive property on the *I–V* characteristics of PSCs, especially on their *I–V* hysteresis phenomena. In theory, the *I–V* hysteresis phenomena are possibly holding the key to further performance optimization of PSCs because of their evident relationship with the *I–V* characteristics of PSCs. As to this assumption, here we also proposed a new device optimization strategy for PSCs based on the analysis results of the effects of memristive property on the *I–V* characteristics of PSCs and memristor theory.

## Results

### Combined model and characteristic equation

**Figure 1:**
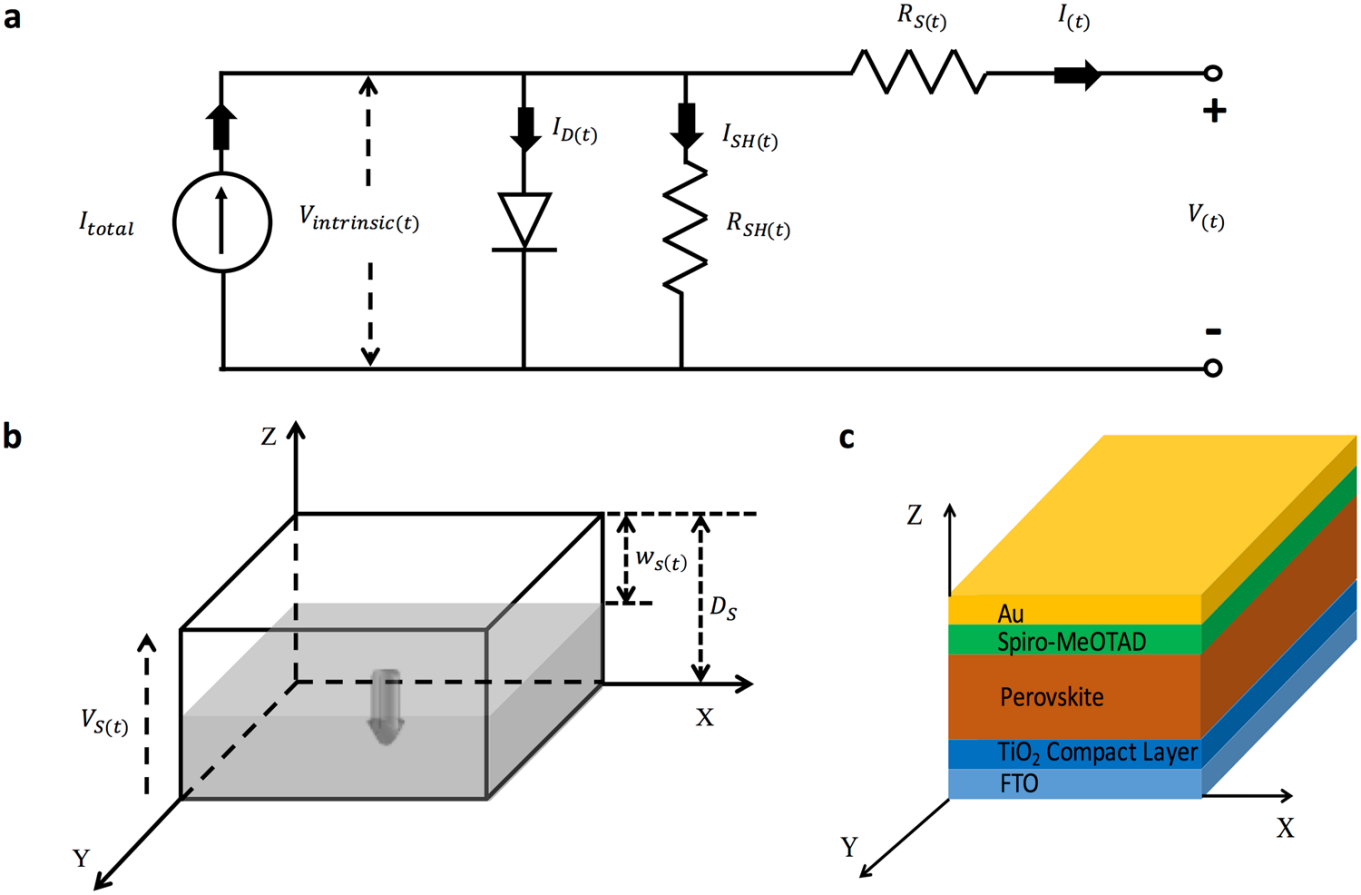
(**a**) The equivalent circuit of a PSC. (**b**) The physical model of the PSC series resistance that was treated as a memristor. The shaded and unshaded areas represent the low-resistance and high-resistance parts, respectively. The arrow denotes the moving direction of the boundary of the two parts under the applied voltage. The physical model of the shunt resistance is similar to that of the series resistance; thus, it is not showed here. (**c**) The common device structure of a PSC. The coordinate used in (**b**) and the one used in (**c)** are linked. The meanings of the marked parameters are listed after the equations below.

To integrate the memristive property into the *I*–*V* characteristics of the PSCs, we started with the combination of their physical models. Figure 1a shows the equivalent circuit for PSC as a widely used physical model for photovoltaic cells in previous studies. Figure 1b illustrates the famous physical model of memristor published in *Nature* in 2008^20^. Series resistance (*R*_*S*(*t*)_) and shunt resistance (*R*_*SH*(*t*)_) were treated as memristors, indicating that their resistance were changeable under the applied voltage during the *I–V* characteristic test. This assumption links the two physical models and enabled us to integrate the memristor parameters into the physical model of PSC. As a result, nine independent equations containing nine independent variables could be obtained as listed below. And due to mathematical principle, these equations could be solved. Based on these nine independent equations (the first five were deduced from the PSC physical model and the last four the memristor physical model), a characteristic equation containing only three variables – *I*_(*t*)_, *V*_(*t*)_ and *t*; the exact corresponding variables in the test for the *I–V* characteristics of PSCs (*t* can be easily overlooked in this test) – can be deduced [Equation (10); the detailed deducing process is shown in Deduction Process S1 Supporting Information].

To be noted, as to the basement of memristive *R*_*S*_ and *R*_*SH*_, namely the mechanism of the memristive property of hybrid perovskites, different reports have concluded various theories, such as charge carrier trapping/de-trapping by defects^17^, variation of the barrier on film interface^19^, low-resistance filament formation^18^ and ion migration^22^. Thus it is still very controversial and further research is needed for determining the exact origin of the memristive property. To ensure the wide adaptability of the joint model and the deduced equations, a general physical model of memristor was intentionally involved in our paper rather than a specific one such as the model based on ion migration theory.

1 $${I}_{total}={I}_{(t)}+{I}_{SH(t)}+{I}_{D(t)}$$

2 $${V}_{intrinsic(t)}={V}_{(t)}+{V}_{S(t)}$$

3 $${I}_{(t)}=\frac{{V}_{S(t)}}{{R}_{S(t)}}$$

4 $${I}_{SH(t)}=\frac{{V}_{intrinsic(t)}}{{R}_{SH(t)}}$$

5 $${I}_{D(t)}={I}_{0}(\exp \frac{{V}_{intrinsic(t)}}{n{V}_{T}}-1)$$

6 $${R}_{S(t)}={R}_{S/OFF}\frac{{w}_{s(t)}}{{D}_{S}}+{R}_{S/ON}(1-\frac{{w}_{s(t)}}{{D}_{S}})$$

7 $$\frac{d{w}_{s(t)}}{dt}={\mu }_{SV}\frac{{V}_{S(t)}}{{D}_{S}}$$

8 $${R}_{SH(t)}={R}_{SH/OFF}\frac{{w}_{SH(t)}}{{D}_{SH}}+{R}_{SH/ON}(1-\frac{{w}_{SH(t)}}{{D}_{SH}})$$

9 $$\frac{d{w}_{SH(t)}}{dt}={\mu }_{SHV}\frac{{V}_{intrinsic(t)}}{{D}_{SH}}$$

10 $$\begin{array}{rcl}{I}_{total} & = & {I}_{(t)}+{I}_{0}(\exp \frac{{V}_{(t)}+{R}_{S(0)}{I}_{(t)}\,\exp (\frac{({R}_{S/OFF}-{R}_{S/ON}){\mu }_{SV}}{{D}_{S}^{2}}{\int }_{0}^{t}{I}_{(t)}dt)}{n{V}_{T}}-1)\\  &  & +\displaystyle \frac{{V}_{(t)}+{R}_{S(0)}\,{I}_{(t)}\,\exp (\frac{({R}_{S/OFF}-{R}_{S/ON}){\mu }_{SV}}{{D}_{S}^{2}}{\int }_{0}^{t}{I}_{(t)}dt)}{\frac{({R}_{SH/OFF}-{R}_{SH/ON}){\mu }_{SHV}}{{D}_{SH}^{2}}{\int }_{0}^{t}[{V}_{(t)}+{R}_{S(0)}{I}_{(t)}\,\exp (\frac{({R}_{S/OFF}-{R}_{S/ON}){\mu }_{SV}}{{{D}_{S}}^{2}}{\int }_{0}^{t}{I}_{(t)}\,dt)]\,dt+{R}_{SH(0)}}\end{array}$$

Here:

- *I*_*total*_: light-generated current [A]
- *I*_(*t*)_: external circuit current at time t [A]
- *I*_*SH*(*t*)_: current flow through the shunt resistance at time t [A]
- *I*_*D*(*t*)_: diode current at time t [A]
- *V*_*intrinsic*(*t*)_: voltage across the constant current source at time t [V]
- *V*_(*t*)_: voltage across the output terminals at time t [V]
- *V*_*S*(*t*)_: voltage across the series resistance at time t [V]
- *R*_*S*(*t*)_(*R*_*SH*(*t*)_): series (shunt) resistance at time t [Ω]
- *I*_0_: reverse saturation current of the diode [A]
- *n*: diode ideality factor
- *V*_*T*_: thermal voltage; $${V}_{T}=\frac{kT}{q}\approx 0.0259\,V$$ (T, 298 K)
- *R*_*S*/*OFF*_(*R*_*SH*/OFF_): off state/low resistance of the series (shunt) resistance [Ω]
- *R*_*S*/*ON*_(*R*_*SH*/*ON*_): on state/high resistance of the series (shunt) resistance [Ω]
- *R*_*S*(0)_ (*R*_*SH*(0)_): initial resistance of the series (shunt) resistance [Ω]
- *w*_*s*(*t*)_(*w*_*SH*(*t*)_): high-resistance area’s length in the series (shunt) resistance at time t [m]
- *D*_*S*_(*D*_*SH*_): length of the series (shunt) resistance along the electric field direction [m]
- *μ*_*SV*_(*μ*_*SHV*_): average ion mobility in the series (shunt) resistance [m^2^s^−1^V^−1^]

### Equation solution

Although Equation (10) is too complicated to have an analytical solution with a form like *I*_(*t*)_ = *f*(*V*_(*t*)_, *t*), obtaining its numerical solution (in this case, a list of *I*_(*t*)_, *V*_(*t*)_, and *t* values that fit the equation within a certain margin of error) is still possible. To obtain the numerical solution of Equation (10), a VBA computation program (shown in Program S1 Supporting Information; it can also be used to fit the actual *I*–*V* data; relative discussion in Figure S7 Supporting Information) within an Excel sheet was designed; the values or expressions (for *V*_(*t*)_) of all parameters can be changed manually to easily investigate their effects on the *I–V* characteristics of PSCs (Supporting Information Excel). The bisection approach was adopted for computation of the numerical solution. But it may not be the most efficient method and further research on the optimal calculation approach is needed to reduce the time consumption of the computation process. The specific values of those constants in Equation (10) were either referred to relative papers^20^ or based on the corresponding graphics. In addition, the diode variable *I*_0_ was treated as a constant here. However, given that it contains various parameters of device properties (Equation S1 Supporting Information), treating it as a variable may be more helpful in the completion of the joint physical model and is suggested for future study.

### Memristive property’s effects on PSCs

**Figure 2:**
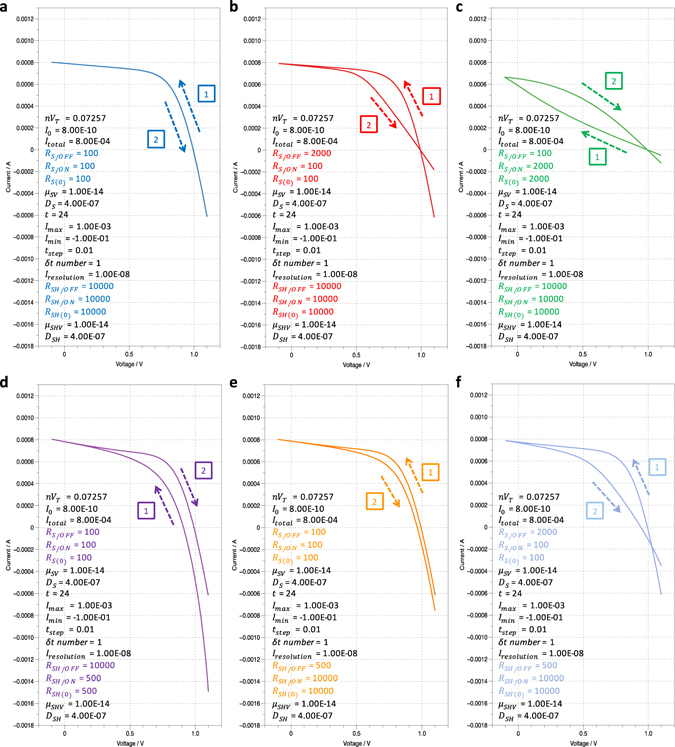
The simulated/computed results of the *I–V* characteristics of PSCs. (**a)** Both *R*_*S*(*t*)_ and *R*_*SH*(*t*)_ show ohmic conduction property. (**b**,**c**) *R*_*SH*(*t*)_ shows ohmic conduction property, whereas *R*_*S*(*t*)_ shows memristive property with resistance that increases and decreases under positive applied voltage. (**d,e**) *R*_*S*(*t*)_ shows ohmic conduction property, whereas *R*_*SH*(*t*)_ shows memristive property with resistance that increases and decreases under positive applied voltage. (**f**) Both *R*_*S*(*t*)_ and *R*_*SH*(*t*)_ show memristive property. The marked numbers and arrows represent the voltage scan sequences and directions, respectively. The specific values of all the parameters used in the simulation/computation are listed in the corresponding charts using SI unit. Except for the colored ones, all others are the same for different cases here.

To investigate the effect of memristive property on the PSCs’ *I–V* characteristics, different sets of parameters was adopted, and six cases was discussed here. As shown in the backward−forward (Fig. 2a–f) and forward−backward voltage scan results (Figure S2a–f, Supporting Information), no *I–V* hysteresis phenomenon was observed when both *R*_*S*(*t*)_ and *R*_*SH*(*t*)_ showed only ohmic conduction property. Other cases that at least one memristive element was in the circuit all showed clear *I–V* hysteresis phenomena. These findings indicated that the *I–V* hysteresis phenomena did result from the memristive property of the materials. By comparing these computed results with the common *I–V* characteristics of PSCs, one can conclude that case b and e were more consistent with the actual test results than case c and d, respectively. This observation means that the resistance of *R*_*S*(*t*)_ tends to increase under positive applied voltage, whereas that of *R*_*SH*(*t*)_ tends to decrease, which may due to the crystal degradation of the function films under the applied voltage. The corresponding *I–V* characteristics when both *R*_*S*(*t*)_ and *R*_*SH*(*t*)_ showed memristive property (*R*_*S*(*t*)_ increased and *R*_*SH*(*t*)_ decreased under positive applied voltage) was simulated based on the above results and showed in Fig. 2f. The simulation result matched very well with the common PSC *I–V* hysteresis phenomenon, indicating the correctness and reliability of our model. As mentioned above, further research is needed to establish the connection between the resistance variation and material change in PSCs. The theory based on ion migration that has been used to explain both the memristive property and *I–V* hysteresis phenomenon may be the key to this challenge. However, due to the diversity of the hybrid perovskite materials and the device structures of perovskite solar cells, a single ion migration theory may not be good enough for all different cases. Thus, utilizing a general physical model rather than a specific one may be a wise choice for investigating the general effects of the memristive property here. However, a more comprehensive model is needed for deeper research in the future, especially when it is applied to a certain type of PSCs.

**Figure 3:**
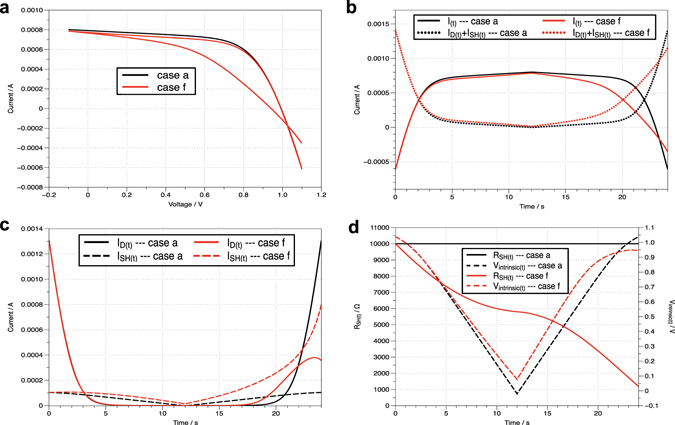
Comparison of the corresponding parameters in cases a and f. (**a)** The *I–V* characteristics of PSCs. **(b**) The change regularities of *I*_(*t*)_ and the sum of *I*_*D*(*t*)_ and *I*_*SH*(*t*)_ over test time (*t*). (**c**) The change regularities of *I*_*D*(*t*)_ and *I*_*SH*(*t*)_ over test time (*t*). (**d**) The change regularities of *R*_*SH*(*t*)_ and *V*_*intrinsic*(*t*)_ over test time (*t*).

The change regularities of the corresponding parameters of cases a (Fig. 2a) and f (Fig. 2f) were compared to further investigate the effects of the memristive property. As shown in Fig. 3a,c, case f showed lower *I*_(*t*)_ and higher sum of *I*_*D*(*t*)_ and *I*_*SH*(*t*)_ than case a. This observation is a natural result because the sum of *I*_(*t*)_, *I*_*D*(*t*)_, and *I*_*SH*(*t*)_ is equal to *I*_*total*_, which is a constant [Equation (1)]. Thus, as shown in Fig. 3b, the higher the sum of *I*_*D*(*t*)_ and *I*_*SH*(*t*)_, the smaller the *I*_(*t*)_. Base on the expressions of *I*_*D*(*t*)_ and *I*_*SH*(*t*)_ [Equations (4) and (5)], the *I*_*D*(*t*)_ value is determined by *V*_*intrinsic*(*t*)_ only, whereas the *I*_*SH*(*t*)_ value is determined by both *V*_*intrinsic*(*t*)_ and *R*_*SH*(*t*)_. The difference in *V*_*intrinsic*(*t*)_ for cases a and f was smaller compared with that of *R*_*SH*(*t*)_, especially for the forward scan part (12–24 s), as displayed in Fig. 3d. Although *R*_*SH*(*t*)_’s value changes of the backward and forward scan parts in case f were similar, the degree of change became increasingly larger as the test time progressed, which resulted in a growing difference of the *I*_*SH*(*t*)_ values of the two cases.

Both the expressions of *I*_*D*(*t*)_ and *I*_*SH*(*t*)_ contain the variable *V*_*intrinsic*(*t*)_ and ${V}_{intrinsic(t)}={V}_{(t)}+{R}_{S(0)}{I}_{(t)}\,\exp$ $(\frac{({R}_{S/OFF}-{R}_{S/ON}){\mu }_{SV}}{{{D}_{S}}^{2}}{\int }_{0}^{t}{I}_{(t)}dt)$. Note that the integral term ${\int }_{0}^{t}{I}_{(t)}dt$ is accompanied by coefficient (*R*_*S*/*OFF*_ − *R*_*S*/*ON*_), thus, when *R*_*S*(*t*)_ shows memristive property, namely, *R*_*S*/*OFF*_ ≠ *R*_*S*/*ON*_, the exponential term on the right of the above equation will not be constant 1. Consequently, the value of the integral term ${\int }_{0}^{t}{I}_{(t)}dt$ will affect the value of *V*_*intrinsic*(*t*)_ (Fig. 3d) and accordingly the values of *I*_*D*(*t*)_ and *I*_*SH*(*t*)_. ${\int }_{0}^{t}{V}_{intrinsic(t)}dt$ is another integral term in Equation (10), which is accompanied by coefficient (*R*_*SH*/*OFF*_ − *R*_*SH*/*ON*_). Thus when *R*_*SH*(*t*)_ shows memristive property, namely, *R*_*SH*/*OFF*_ ≠ *R*_*SN*/*ON*_, the value of the integral term ${\int }_{0}^{t}{V}_{intrinsic(t)}dt$ will affect the values of other corresponding parameters in Equation (10). In contrast, when both *R*_*S*(*t*)_ and *R*_*SH*(*t*)_ showed only ohmic conduction property, the coefficients of ${\int }_{0}^{t}{I}_{(t)}dt$ and ${\int }_{0}^{t}{V}_{intrinsic(t)}dt$ will be 0, resulting in 0 effects of the two integral term on the *I–V* characteristics of PSCs. This is the physical basis of the identical *I*–*V* curves of Figs 2a and S2a that both showed no *I–V* hysteresis phenomenon. Thus it indicated that the memristive property is the origin of the *I–V* hysteresis of PSCs.

The above-mentioned two integral term – ${\int }_{0}^{t}{I}_{(t)}dt$ and ${\int }_{0}^{t}{V}_{intrinsic(t)}dt$ – are the exact parameters whose values are connected with the specific *I–V* characteristics test conditions, such as the voltage scan direction, voltage scan speed, and others. For example, the value of ${\int }_{0}^{t}{I}_{(t)}dt$ at the begging of the forward-scan in a forward-backward scan test will be 0; but it isn’t 0 in a backward-forward scan test; the above-mentioned difference thus endows the *I–V* characteristics of PSCs with dependence on the scan direction of the *I*–*V* test. The two integral terms endow the *I–V* characteristics of PSCs with dependence on the test method/process, which is a widely observed fact for PSCs; the severity of the corresponding effects are determined by the specific memristive property of the perovskite material (mainly through the above-mentioned two coefficients). Furthermore, from a physical/mathematical point of view, these two integral terms ensure that “*I*_(*t*)_ versus *t*” is continuous and differentiable at every part of itself, and no sudden value change of *I*_(*t*)_ occurs after the voltage scan direction changes in the continuous voltage scan mode (Figure S3 Supporting Information). To be noted, the physical definition of ${\int }_{0}^{t}{I}_{(t)}dt$ is evident, that is, the charge that flows though the external circuit. As to ${\int }_{0}^{t}{V}_{intrinsic(t)}dt$, although its unit is same to that of magnetic flux, its actual physical meaning in the model is vague and thus should be further investigated.

**Figure 4:**
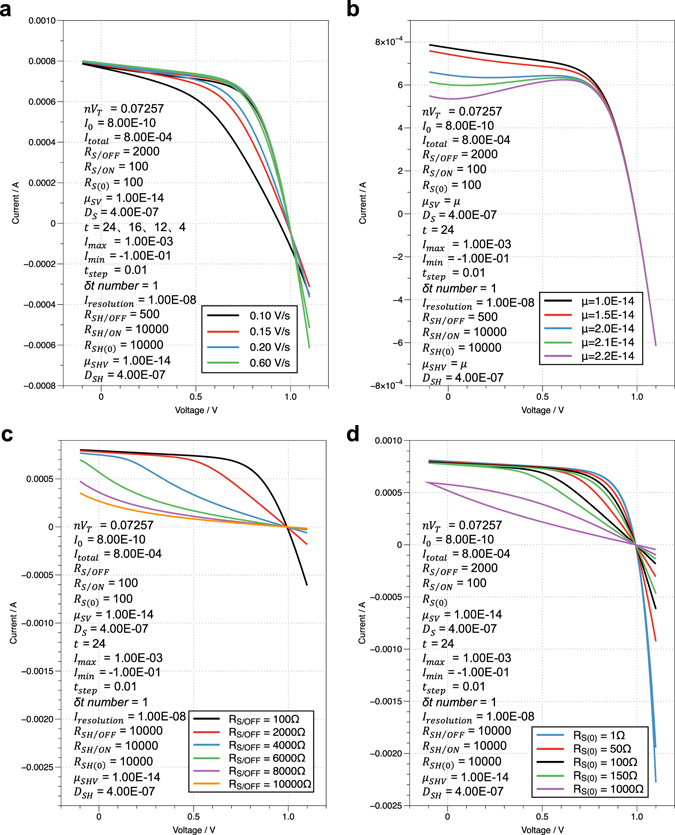
(**a**) The simulated/computed results of the dependence of the *I–V* characteristics of PSCs on voltage scan speed. (**b**) The simulated/computed results of the “bumping curve” phenomenon in the backward voltage scan. The “bumping curve” is realized by adjusting the values of *μ*_*SV*_ and *μ*_*SHV*_, which are endowed with the same value for simplicity. (**c**) The simulated/computed results of the “inward curve” phenomenon in the forward voltage scan. The “inward curve” is realized by adjusting the *R*_*S*/*OFF*_ value. The *R*_*SH*_ is treated as an ohmic resistance for simplicity. (**d**) The simulated/computed results of the *I–V* characteristics of PSCs with different *R*_*S*(0)_. The *R*_*SH*_ is treated as an ohmic resistance for simplicity. The specific values of all the parameters used in the simulation/computation are listed in the corresponding charts using SI unit.

Equation (10) was found to be a powerful tool for PSC research and could also be used to simulate and explain numerous *I–V* characteristics of PSCs, for example, the dependence of the *I*–*V* characteristics on voltage scan speed (Fig. 4a), “bumping curve” phenomena in the backward voltage scan (Fig. 4b), and “inward curve” phenomena in the forward voltage scan (Fig. 4c). There’re few studies reported on the last two phenomena, not mention their theoretical-level analysis or simulation; thus the findings presented here may be instructive for further corresponding study. Using the similar analysis process mentioned above in comparing cases a and f, one can deduce the origins of these phenomena (Figures S4–S6 Supporting Information). All these results indicated that the memristive is the origin of the unique *I–V* characteristics of PSCs and proved the considerable potential of Equation (10) for various further research on PSCs. One important aspect of the theoretical research is to fit the actual *I*–*V* characteristics of PSCs using the joint model and deduced equation. One fitting result along with the introduction of the corresponding computation method is shown in Figure S7 Supporting Information. The reasonable fitting result indicated the correctness and practicability of the deduced equation. To get a more satisfactory fitting result, several strategies can be adopted, such as decreasing the computation-size or increasing the computation-scope of the corresponding simulation variables and adding more parameters as variables. Either way, however, would significantly increase the computation time. And due to the dozens of variables in the equation, obtaining a satisfactory fitting result in short computation time was found to be quite challenging. Thus, as mentioned above, further research on the optimal calculation approach is needed to reduce the time consumption of the computation/fitting process.

Aside from its function to simulate and analyze the *I–V* characteristics of PSCs, a perhaps more important inspiration we obtained based on Equation (10) was a new device optimization strategy for PSCs. As we know, memristor’s resistance is changeable and can be easily adjusted using applied voltage. What if we can adjust the resistance of the memristive elements in PSCs? Will it help to improve the device performance? As shown in Fig. 4d, the device displays improved performance when *R*_*S*(0)_ is “adjusted” to a lower value, and if *R*_*S*(0)_ is lower than *R*_*S*/*ON*_, the device performance will be better. To realize this, one can refer to those methods to deduce the on-state resistance or increase the low-resistance current in the field of memristors. This attempt may help link the two areas and may lead to some unexpected positive results. Besides, based on the above result, the application of pretest treatment in the *I–V* characteristics test of PSCs, which has been adopted by numerous reported studies, may result in the value changes of *R*_*S*(0)_ and *R*_*SH*(0)_ (and the two integral terms in Equation (10) as well; a relaxation time may be possible for the corresponding effects) and accordingly affect the apparent device performance. Thus, the standardization of the *I–V* characteristics test method is indeed very necessary, as we mentioned in the Introduction.

## Discussion

We introduced the memristive property into the analysis of the *I*–*V* characteristics of PSCs and the results showed that the memristive property is the origin of various types of *I*–*V* characteristics of PSCs. The obtained joint model and the deduced equation may help solving the long-existent mysteries of the *I–V* characteristics of PSCs and the proposed device optimization strategy may further develop into a novel approach for increasing the performance of PSCs to the limit. Based on the results of this research and the fact that memristive behaviors are very sensitive to the applied voltage and the time of voltage treatment, we propose suggestions as followed for improving the accuracy of measuring the intrinsic device performance of PSCs: 1. any pre-test treatments that will affect the memristive behaviors of the hybrid perovskites should be clarified; 2. the range of the voltage scan, the voltage step size and the step time (not just the scan speed) of the *I*–*V* characteristics test of PSCs should be unified; 3. in long-term stability test, any changes of the *I*–*V* hysteresis phenomena should also be presented to give an evaluation of the memristive effects that accumulated over the test time.

There’re many reported high-performance perovskite solar cells with unnoticeable *I*–*V* hysteresis^4, 23^, which are usually ascribed to the utilization of perovskite function layer with high crystallinity and few defects. With better film-quality, the corresponding part may show less severe memristive behavior, considering that many mechanisms of the memristive property are based on crystal defects^24^. Thus, by optimizing the film-quality of perovskite solar cells, the memristive behavior of the corresponding part will be suppressed and it will result in less severe *I*–*V* hysteresis phenomenon. Figure 2a shows the no-hysteresis *I*–*V* curve of an ideal PSC whose *R*_*S*_ and *R*_*SH*_ are both ideal ohmic resistance; this however may not be practical as for real PSCs because of the unavoidable crystal defects in function layers. Even for those high-performance perovskite solar cells with unnoticeable *I*–*V* hysteresis in the initial stage, certain film degradation may occur after long-term usage and it may lead to the enhanced memristive behaviors and accordingly the increasingly-clear *I*–*V* hysteresis phenomenon in the end. Further research is needed to elucidate this accumulated effects of the memristive effects.

As to the specific memristive mechanism of hybrid perovskites, different reports have concluded various theories, such as charge carrier trapping/de-trapping by defects^17^, variation of the barrier on film interface^19^, low-resistance filament formation^18^ and ion migration^22^; thus further research is needed for determining the exact origin of the memristive property. To ensure the wide adaptability of the joint model and the deduced equation, a general physical model of memristor was intentionally involved in this research rather than a specific one. But a more specific model is needed for deeper research in the future, especially when it is applied to a certain type of PSCs. Meanwhile, a more comprehensive and accurate simulation can be expected with the introduction of more complicated PSCs and memristor physical models into the joint one. For example, a capacitor element can be added to PSC physical model as needed or *I*_0_ can be considered as a variable. In addition to our primary discussions and results, various further research can be conducted on the basis of the proposed model and equations, and a deeper understanding of the effects of the memristive property on PSCs can be expected.

### Data Availability Statement

The datasets generated during and/or analyzed during the current study are available from the corresponding author on reasonable request.

## Electronic supplementary material

Supplementary Dataset

Supporting Information
