# Supplementary material for: Memristive property’s effects on the I–V characteristics of perovskite solar cells
Source: Sci Rep. 2017 Jul 20;7:6025. doi: 10.1038/s41598-017-05508-5 (PMC5519685; doi:10.1038/s41598-017-05508-5)
Supplement: Supplementary file 2 — Supporting Information [file 41598_2017_5508_MOESM2_ESM.docx]

**Supporting Information**

**Memristive property's effects on the *I–V* characteristics of perovskite solar cells**

*Kai Yan, Bin Dong, Xinyu Xiao, Si Chen, Buxin Chen, Xue Gao, Hsienwei Hu, Wen Wen, Jingbo Zhou, Dechun Zou ^*^*

**An Example of manipulating the device performance of the perovskite solar cells**

**Figure S1** The *I-V* characteristics of a perovskite solar cell under different testing methods. The open circuit voltage (*V_OC_*) of the backward scan can be manipulated by fast scanning from different starting voltages. The black line represents the *I−V* characteristics during the normal test and the other lines denote the *I−V* characteristics during the fast scanning method. The starting voltage, voltage step size, step time, and scan speed of this procedure are illustrated in the inset. These “exaggerated” results were intentional chosen to show the unreliability of the tests, that is, the highest *V_OC_* is considerably higher than the theoretical limit. Any particular power conversion efficiencies can be achieved because the short circuit current can be manipulated by changing the light illumination condition (e.g., the distance between a light source and the solar cell) and the “artificial” *V_OC_*.

The corresponding device used in the test was a common planar heterojunction perovskite solar cell with the structure as “FTO/TiO_2_-compact layer/CH_3_NH_3_PbI*_X_*Cl_3-_*_X_*/Spiro-MeOTAD/Au”. The function layers of the device was fabricated using the same method in previous report^1^.

**Equation Deduction Process**

Using Equation (2)–(5) to replace the relative parts in Equation (1), we can obtain the following:

$I_{total}=I_{\left( t \right)}+I_{0}\left( exp\frac{V_{\left( t \right)}+V_{S\left( t \right)}}{nV_{T}}-1 \right)+\frac{V_{\left( t \right)}+V_{S\left( t \right)}}{R_{SH(t)}}$ (11)

Equation (10) contains $I_{\left( t \right)}$, $V_{\left( t \right)}$, $V_{S\left( t \right)}$ and $R_{SH(t)}$; thus, we have to replace variables $V_{S\left( t \right)}$ and $R_{SH(t)}$ with expressions containing only $I_{\left( t \right)}$ and/or $V_{\left( t \right)}$ and/or $t$ to obtain the equation with a form as $I_{\left( t \right)}=f\left( V_{\left( t \right)}, t \right)$.

On the basis of Equation (6), we can derive the following:

$\frac{dR_{\left. S(t \right)}}{dt}=\frac{\left( R_{S/OFF}-R_{S/ON} \right)}{D_{S}}\frac{dw_{S\left. (t \right)}}{dt}$ (12)

Given Equations (7) and (12), we can obtain the following:

$\frac{dR_{\left. S(t \right)}}{dt}=\frac{\left( R_{S/OFF}-R_{S/ON} \right)\mu_{SV}I_{\left( t \right)}R_{\left. S(t \right)}}{{D_{S}}^{2}}$ (13)

Using Equation (13), we can obtain the following:

$\frac{1}{R_{\left. S(t \right)}}dR_{\left. S(t \right)}=\frac{\left( R_{S/OFF}-R_{S/ON} \right)\mu_{SV}}{{D_{S}}^{2}}I_{\left( t \right)}dt$ (14)

Integrating both sides of Equation (14), we can obtain the following:

${lnR}_{\left. S(t \right)}-{lnR}_{\left. S(0 \right)}=\frac{\left( R_{S/OFF}-R_{S/ON} \right)\mu_{SV}}{{D_{S}}^{2}}\int_{0}^{t} I_{\left( t \right)}dt$ (15)

Based on Equation (15), we can obtain the following:

$R_{\left. S(t \right)}=R_{\left. S(0 \right)}exp\left( \frac{\left( R_{S/OFF}-R_{S/ON} \right)\mu_{SV}}{{D_{S}}^{2}}\int_{0}^{t} I_{\left( t \right)}dt \right)$ (16)

Using Equation (16), we can derive the following:

$V_{S\left( t \right)}=I_{\left( t \right)}R_{\left. S(t \right)}=R_{\left. S(0 \right)}I_{\left( t \right)}exp\left( \frac{\left( R_{S/OFF}-R_{S/ON} \right)\mu_{SV}}{{D_{S}}^{2}}\int_{0}^{t} I_{\left( t \right)}dt \right)$ (17)

On the basis of Equation (9), we can obtain the following:

$dw_{SH\left( t \right)}=\mu_{SHV}\frac{V_{intrinsic(t)}}{D_{SH}}dt$ (18)

Integrating both sides of Equation (18), we can obtain the following:

$w_{SH\left( t \right)}-w_{SH\left( 0 \right)}=\int_{0}^{t} \mu_{SHV}\frac{V_{intrinsic(t)}}{D_{SH}}dt=\frac{\mu_{SHV}}{D_{SH}}\int_{0}^{t} \left( V_{\left( t \right)}+V_{S\left( t \right)} \right)dt$ (19)

Using Equation (17) and (19), we can obtain the following:

$w_{SH\left( t \right)}=\frac{\mu_{SHV}}{D_{SH}}\int_{0}^{t} \left[ V_{\left( t \right)}+R_{\left. S(0 \right)}I_{\left( t \right)}exp\left( \frac{\left( R_{S/OFF}-R_{S/ON} \right)\mu_{SV}}{{D_{S}}^{2}}\int_{0}^{t} I_{\left( t \right)}dt \right) \right]dt+w_{SH\left( 0 \right)}$ (20)

With Equation (8) and (20), we can obtain the following:

$R_{SH(t)}=\frac{\left( R_{{SH}/OFF}-R_{{SH}/ON} \right)\mu_{SHV}}{{D_{SH}}^{2}}\int_{0}^{t} \left[ V_{\left( t \right)}+R_{\left. S(0 \right)}I_{\left( t \right)}exp\left( \frac{\left( R_{S/OFF}-R_{S/ON} \right)\mu_{SV}}{{D_{S}}^{2}}\int_{0}^{t} I_{\left( t \right)}dt \right) \right]dt+R_{\left. SH(0 \right)}$ (21)

Given Equation (17), (21) and (11), one can get equation (10) which includes only three key variables, namely $I_{\left( t \right)}$, $V_{\left( t \right)}$ and *t*.

PS: $V_{\left( t \right)}$, which is equal to the applied voltage of the *I–V* characteristics test of PSCs, has not been provided with any specific restrictions yet until this step. It denotes that the *I–V* characteristics of PSCs under commonly used linear and unconventional voltage scan modes (e.g., sinusoidal wave model) can be both investigated using Equation (10), making this equation a powerful tool for future study on PSCs.

**VBA Program in Excel for computing the numerical solution of Equation (10) (The expressions of variables may be slightly different from their counterparts in the equations for convenience in writing in the program)**

Option Explicit

Dim nVt As Double

Dim I0 As Double

Dim Itotal As Double

Dim Rsoff As Double

Dim Rson As Double

Dim Rs0 As Double

Dim usv As Double

Dim Ds As Double

Dim Time As Double

Dim tstep As Double

Dim dtNumber As Long

Dim Vintr As Double

Dim Icumu As Double

Dim Vcumu As Double

Dim Imax, Imin, Iresolution As Double

Dim Rshoff As Double

Dim Rshon As Double

Dim Rsh0 As Double

Dim ushv As Double

Dim Dsh As Double

Dim Vt As Double

Dim IDt As Double

Dim Isht As Double

Dim Rst As Double

Dim Rst1 As Double

Dim wst As Double

Dim Rsht As Double

Dim Rsht1 As Double

Dim wsht As Double

Dim Data(1 To 100000, 1 To 12) As Double

Dim result(1 To 100000, 1 To 20) As Double

Dim varNo(1 To 100) As Integer, varNumber As Integer

Dim varStart(1 To 100) As Double, varEnd(1 To 100) As Double, varStep(1 To 100) As Double, varResult(1 To 100) As Double

Dim count As Long, countFit As Long, sum As Double, minSum As Double, minIndex As Long

Dim rowCount As Integer, dataRowCount As Integer, varRowCount As Integer

Public Sub StartCalc()

Dim i As Integer, j As Integer

Sheets(1).Activate

nVt = Cells(2, "B").value

I0 = Cells(3, "B").value

Itotal = Cells(4, "B").value

Rsoff = Cells(5, "B").value

Rson = Cells(6, "B").value

Rs0 = Cells(7, "B").value

usv = Cells(8, "B").value

Ds = Cells(9, "B").value

Time = Cells(10, "B").value

Imax = Cells(11, "B").value

Imin = Cells(12, "B").value

tstep = Cells(13, "B").value

dtNumber = Cells(14, "B").value

Iresolution = Cells(15, "B").value

Rshoff = Cells(16, "B").value

Rshon = Cells(17, "B").value

Rsh0 = Cells(18, "B").value

ushv = Cells(19, "B").value

Dsh = Cells(20, "B").value

minSum = 1E+100

countFit = 1

Erase Data

Erase result

rowCount = Range("C1048576").End(xlUp).Row

varRowCount = Range("E1048576").End(xlUp).Row

dataRowCount = Sheets(2).Range("A1048576").End(xlUp).Row

varNumber = CInt((varRowCount - 1) / 5)

For i = 1 To varNumber Step 1

varNo(i) = Cells((i - 1) * 5 + 2, "F").value

varStart(i) = Cells((i - 1) * 5 + 3, "F").value

varEnd(i) = Cells((i - 1) * 5 + 4, "F").value

varStep(i) = Cells((i - 1) * 5 + 5, "F").value

Next

If Cells(1, "G").value <> 0 Then

VarTrial 1

For i = 1 To varNumber Step 1

SetVal varNo(i), result(minIndex, i)

Next

End If

Calculation False

Columns("I:T").Clear

Sheets(2).Range(Sheets(2).Cells(1, "D"), Sheets(2).Cells(dataRowCount, "Z")).Clear

Columns("I:T").ColumnWidth = 8

Columns("I:T").Font.Name = "Times New Roman"

Columns("I:T").Font.Size = 12

Cells(1, "I").value = "t"

Cells(1, "J").value = "V(t)"

Cells(1, "J").Characters(Start:=2, Length:=3).Font.Subscript = True

Cells(1, "K").value = "I(t)"

Cells(1, "K").Characters(Start:=2, Length:=3).Font.Subscript = True

Cells(1, "L").value = "ID(t)"

Cells(1, "L").Characters(Start:=2, Length:=4).Font.Subscript = True

Cells(1, "M").value = "ISH(t)"

Cells(1, "M").Characters(Start:=2, Length:=5).Font.Subscript = True

Cells(1, "N").value = "Icumu(t)"

Cells(1, "N").Characters(Start:=2, Length:=7).Font.Subscript = True

Cells(1, "O").value = "Vcumu(t)"

Cells(1, "O").Characters(Start:=2, Length:=7).Font.Subscript = True

Cells(1, "P").value = "RS(t)"

Cells(1, "P").Characters(Start:=2, Length:=4).Font.Subscript = True

Cells(1, "Q").value = "wS(t)"

Cells(1, "Q").Characters(Start:=2, Length:=4).Font.Subscript = True

Cells(1, "R").value = "RSH(t)"

Cells(1, "R").Characters(Start:=2, Length:=5).Font.Subscript = True

Cells(1, "S").value = "wSH(t)"

Cells(1, "S").Characters(Start:=2, Length:=5).Font.Subscript = True

Cells(1, "T").value = "Vintr(t)"

Cells(1, "T").Characters(Start:=2, Length:=7).Font.Subscript = True

Range("I1:T1").HorizontalAlignment = xlRight

Sheets(2).Range("D1:Z1").HorizontalAlignment = xlRight

For i = 1 To varNumber Step 1

If varNo(i) >= 2 And varNo(i) <= 20 Then Sheets(2).Cells(1, 3 + i) = Cells(varNo(i), 1)

Cells((i - 1) * 5 + 6, "F").value = result(minIndex, i)

Next

Sheets(2).Cells(1, 4 + varNumber).value = "Sum"

Cells(2, "I").Resize(count, 12) = Data

If countFit > 1 Then

Sheets(2).Cells(2, "D").Resize(countFit - 1, varNumber + 1) = result

End If

End Sub

Public Sub VarTrial(currentIndex As Integer)

Dim val As Double

Dim thisVarNo As Integer

If currentIndex > varNumber Then

Calculation True

Exit Sub

End If

thisVarNo = varNo(currentIndex)

If thisVarNo < 2 Or thisVarNo > 20 Then

VarTrial currentIndex + 1

Else

For val = varStart(currentIndex) To varEnd(currentIndex) Step varStep(currentIndex)

SetVal thisVarNo, val

varResult(currentIndex) = val

VarTrial currentIndex + 1

Next

End If

End Sub

Public Sub Calculation(flag As Boolean)

Dim I1 As Double, I2 As Double, Imid As Double

Dim value1, value2, valueMid As Double

Dim Vstring As String

Dim index As Integer

Dim tstepInner As Double

Dim i As Integer, j As Integer

Dim t As Double, t1 As Double

Icumu = 0

Vcumu = 0

count = 0

sum = 0

For t = 0 To Time Step tstep

For i = 2 To rowCount Step 1

If Cells(i, "C").value > t Then

Exit For

End If

Next

Vstring = Cells(i - 1, "D").value

index = InStr(1, Vstring, "t", vbTextCompare)

If index > 0 Then

Vstring = Mid(Vstring, 1, index - 1) + Str(t) + Mid(Vstring, index + 1)

End If

Vt = Evaluate(Vstring)

I1 = Imin

I2 = Imax

Imid = (I1 + I2) / 2

Do While Abs(I1 - I2) > Iresolution

value1 = Ivalue(I1)

value2 = Ivalue(I2)

valueMid = Ivalue(Imid)

If value1 * value2 < 0 Then

If value1 * valueMid < 0 Then

I2 = Imid

Else

I1 = Imid

End If

ElseIf Abs(value1) < Abs(value2) Then

Imid = I1

Exit Do

Else

Imid = I2

Exit Do

End If

Imid = (I1 + I2) / 2

Loop

value1 = Ivalue(Imid)

count = count + 1

Data(count, 1) = t

Data(count, 2) = Vt

Data(count, 3) = Imid

Data(count, 4) = IDt

Data(count, 5) = Isht

Data(count, 6) = Icumu

Data(count, 7) = Vcumu

Data(count, 8) = Rst

Data(count, 9) = wst

Data(count, 10) = Rsht

Data(count, 11) = wsht

Data(count, 12) = Vintr

Icumu = Icumu + Imid * tstep

Vcumu = Vcumu + Vintr * tstep

If dtNumber > 0 Then

tstepInner = tstep / dtNumber

For t1 = t + tstepInner To t + tstep - tstepInner Step tstepInner

I1 = Imin

I2 = Imax

Imid = (I1 + I2) / 2

Do While Abs(I1 - I2) > Iresolution

value1 = Ivalue(I1)

value2 = Ivalue(I2)

valueMid = Ivalue(Imid)

If value1 * value2 < 0 Then

If value1 * valueMid < 0 Then

I2 = Imid

Else

I1 = Imid

End If

ElseIf Abs(value1) < Abs(value2) Then

Imid = I1

Exit Do

Else

Imid = I2

Exit Do

End If

Imid = (I1 + I2) / 2

Loop

value1 = Ivalue(Imid)

count = count + 1

Data(count, 1) = t1

Data(count, 2) = Vt

Data(count, 3) = Imid

Data(count, 4) = IDt

Data(count, 5) = Isht

Data(count, 6) = Icumu

Data(count, 7) = Vcumu

Data(count, 8) = Rst

Data(count, 9) = wst

Data(count, 10) = Rsht

Data(count, 11) = wsht

Data(count, 12) = Vintr

Icumu = Icumu + Imid * tstepInner

Vcumu = Vcumu + Vintr * tstepInner

Next

End If

Next

If Not flag Then

Exit Sub

End If

j = 1

For i = 1 To dataRowCount Step 1

If Sheets(2).Cells(i, 1).value >= 0 And Sheets(2).Cells(i, 2).value >= 0 Then

While Abs(Data(j, 2) - Sheets(2).Cells(i, 1).value) > 0.0001

j = j + 1

Wend

sum = sum + (Sheets(2).Cells(i, 2).value - Data(j, 3)) * _

(Sheets(2).Cells(i, 2).value - Data(j, 3))

End If

Next

For i = 1 To varNumber Step 1

result(countFit, i) = varResult(i)

Next

result(countFit, varNumber + 1) = sum

If sum < minSum Then

minSum = sum

minIndex = countFit

End If

countFit = countFit + 1

End Sub

Public Function Ivalue(It As Double)

Dim result As Double

Rst = Rs0 * Exp((Rsoff - Rson) * usv / (Ds ^ 2) * Icumu)

If Rst < 0 Then Rst = Rst1

If Rsoff = Rson Then

wst = 0

Else

wst = (Rst - Rson) / (Rsoff - Rson) * Ds

End If

If wst < 0 Then

'wst = 0

'Rst = Rson

ElseIf wst > Ds Then

wst = Ds

Rst = Rsoff

End If

Rsht = (Rshoff - Rshon) * ushv / (Dsh ^ 2) * Vcumu + Rsh0

If Rsht < 0 Then Rsht = Rsht1

If Rshoff = Rshon Then

wsht = 0

Else

wsht = (Rsht - Rshon) / (Rshoff - Rshon) * Dsh

End If

If wsht < 0 Then

'wsht = 0

'Rsht = Rshon

ElseIf wsht > Dsh Then

wsht = Dsh

Rsht = Rshoff

End If

Vintr = Vt + Rst * It

IDt = I0 * (Exp(Vintr / nVt) - 1)

Isht = Vintr / Rsht

Rst1 = Rst

Rsht1 = Rsht1

result = It + IDt + Isht

Ivalue = Itotal - result

End Function

Public Sub SetVal(valNo As Integer, newValue As Double)

Select Case valNo

Case 2

nVt = newValue

Case 3

I0 = newValue

Case 4

Itotal = newValue

Case 5

Rsoff = newValue

Case 6

Rson = newValue

Case 7

Rs0 = newValue

Case 8

usv = newValue

Case 9

Ds = newValue

Case 10

Time = newValue

Case 11

Imax = newValue

Case 12

Imin = newValue

Case 13

tstep = newValue

Case 14

dtNumber = newValue

Case 15

Iresolution = newValue

Case 16

Rshoff = newValue

Case 17

Rshon = newValue

Case 18

Rsh0 = newValue

Case 19

ushv = newValue

Case 20

Dsh = newValue

End Select

End Sub

**Equation of the reverse saturation current of the diode** $\boldsymbol{I}_{\boldsymbol{0}}$

$$I_{0}=qA\left( \sqrt{\frac{D_{p}}{\tau_{p}}}\frac{n_{i}^{2}}{N_{D}}+\sqrt{\frac{D_{n}}{\tau_{n}}}\frac{n_{i}^{2}}{N_{A}} \right)$$

Here:

$q$: the elementary charge

$A$: the cross-sectional area

$D_{p, n}$: the diffusion coefficients of holes and electrons, respectively

$N_{D, A}$: the donor and acceptor concentrations at the n side and p side, respectively

$n_{i}$: the intrinsic carrier concentration in the semiconductor material

$\tau_{p, n}$: the carrier lifetimes of holes and electrons, respectively

**Simulated/computed forward-backward voltage scan results**

**Figure S2** The simulated/computed results of the *I−V* characteristics of PSCs. **(a)** Both $R_{\left. S(t \right)}$ and $R_{\left. SH(t \right)}$ show ohmic conduction property. **(b, c)** $R_{\left. SH(t \right)}$ shows the ohmic conduction property and $R_{\left. S(t \right)}$ shows the memristive property whose resistance increases and decreases at a positive applied voltage. **(d, e)** $R_{\left. S(t \right)}$ shows the ohmic conduction property and $R_{\left. SH(t \right)}$ shows the memristive property whose resistance increases and decreases at a positive applied voltage. **(f)** Both $R_{\left. S(t \right)}$ and $R_{\left. SH(t \right)}$ show memristive property. The marked numbers and arrows denote the voltage scan sequences and directions, respectively. The specific values of all the parameters used in the simulation/computation are listed in the corresponding charts by using SI unit. Except for the colored ones, all others are the same for different cases here.

**Effects of the integral terms –** $\int_{\boldsymbol{0}}^{\boldsymbol{t}} \boldsymbol{V}_{\boldsymbol{intrinsic}\boldsymbol{(}\boldsymbol{t}\boldsymbol{)}}\boldsymbol{dt}$ **and** $\int_{\boldsymbol{0}}^{\boldsymbol{t}} \boldsymbol{I}_{\left( \boldsymbol{t} \right)}\boldsymbol{dt}$

**Figure S3** The simulated/computed results of the *I−V* characteristics of PSCs through continuous (solid black curve) and single backward/forward voltage scans. The specific values of the parameters used in the simulation/computation are listed in the chart using SI unit. No sudden changes of $I_{\left( t \right)}$ occurred after the voltage scan direction changed ($V_{\left( t \right)}=-0.1 V$) in the continuous voltage scan mode. However, $I_{\left( t \right)}$ at −0.1 V differed for single backward/forward voltage scan modes. This difference is attributed to the two integral terms in Equation (10). At the start of the forward scan, the value of the two integral terms is and is not 0 as for the single forward scan model and the continuous scan mode, respectively.

**Analysis of the dependence of the *I-V* characteristics on voltage scan speed**

**Figure S4** The simulated/computed results of the dependence of the *I−V* characteristics of PSCs on voltage scan speed. **a, b, c, d** Experimental groups; all of the parameters are similar to those in case **f** except the total test time $t$. Same parameters were used for the curves with same color. As shown in **d**, the experimental group under the faster scan speed showed fewer changes in $R_{\left. SH(t \right)}$ and $V_{intrinsic(t)}$. The two parameters directly affect $I_{D(t)}$ and $I_{SH(t)}$. Thus, As shown in **c**, the curves of $I_{D(t)}$ and $I_{SH(t)}$ showed less difference between the backward and forward scan parts. Hence, it was same to the sum of $I_{D(t)}$ and $I_{SH(t)}$ and accordingly $I_{(t)}$ between the backward and forward scan parts, as shown in **b**. The final effect was a less severe *I−V* hysteresis phenomenon at a faster scan speed, as shown in **a**. **e** The control group; all of the parameters are similar to those in case **a** except the total test time $t$. The dependence of the *I−V* characteristics of PSCs on voltage scan speed was eliminated when $R_{\left. S(t \right)}$ and $R_{\left. SH(t \right)}$ showed only ohmic conduction property. These results indicated that the memristive property in PSCs caused the dependence of their *I−V* characteristics on voltage scan speed. Thus, to suppress this unfavorable property, we should eliminate or reduce the memristive property in PSCs; for example, immobilizing mobile ions in the perovskite layer (if we assume that the memristive property is due to the mobile ions). The effect may be the reduced $\mu_{SV}$ and $\mu_{SHV}$, and a corresponding simulated result is shown in **f**. The improvement of the *I-V* characteristics of the PSCs was very evident.

**Analysis of the “bumping curve” phenomena in the backward voltage scan**

**Figure S5** The simulated/computed results of the “bumping curve” phenomenon in the backward voltage scan. **a** Simulated *I−V* characteristics of PSCs with different $\mu_{SV}$and $\mu_{SHV}$. **b** Change regularities of $I_{\left( t \right)}$ and sum of $I_{D(t)}$ and $I_{SH(t)}$ over test time $t$. **c** Change regularities of $I_{D(t)}$ and $I_{SH(t)}$ over test time $t$. **(d)** Change regularities of $R_{\left. SH(t \right)}$ and $V_{intrinsic(t)}$ over test time $t$. Only the case colored black that showed no “bumping curve” phenomenon and the case colored purple that showed “bumping curve” phenomenon were compared for simplicity. $\mu_{SV}$and $\mu_{SHV}$ were endowed with similar value for simplicity’s sake, and all other parameters used in the simulation are similar to those in case **f**. The curves with the same color in different figures are the relative results with the same set of simulation parameters. As shown in **d**, $R_{\left. SH(t \right)}$ of the case colored purple decreased faster than that of the case colored black over the test time $t$; and their $V_{intrinsic(t)}$ was almost the same. Thus, these two cases showed similar $I_{D(t)}$ but different $I_{SH(t)}$. The rapid increase in $I_{SH(t)}$ of the case colored purple in the latter part of the scan caused the sum of $I_{D(t)}$ and $I_{SH(t)}$ to increase. As a result, $I_{\left( t \right)}$ decreased in the corresponding part as shown in **b** and thus caused the “bumping curve” phenomenon as shown in **a**.

**Analysis of the “inward curve” phenomena in the forward voltage scan**

**Figure S6** The simulated/computed results of the “inward curve” phenomena in the forward voltage scan. **a** Simulated *I−V* characteristics of the PSCs with different $R_{S/OFF}$. $R_{SH(t)}$ was treated as an ohmic resistance for simplicity. If $R_{S/OFF}=100 \Omega$, $R_{S(t)}$ became an ohmic resistance and thus was marked as $R_{S}$. **b** Change regularities of $I_{\left( t \right)}$ and sum of $I_{D(t)}$ and $I_{SH(t)}$ over test time $t$. **c** Change regularities of $I_{D(t)}$ and $I_{SH(t)}$ over test time $t$. **d** Change regularity of $V_{intrinsic(t)}$ over test time $t$. Only the case colored black that showed no “inward curve” phenomenon and the case colored green that showed clear “inward curve” phenomena were compared for simplicity. The curves with the same color in different figures are relative results using the same set of simulation parameters. Because $R_{SH(t)}$ was treat as an ohmic resistance here, $V_{intrinsic(t)}$ became the only parameter that affect $I_{D(t)}$ and $I_{SH(t)}$ directly. As shown in **d**, the case colored green showed higher $V_{intrinsic(t)}$ than the case colored black in the first part of the forward scan. As a result, a considerably higher $I_{D(t)}$ was obtained for the case colored green, as shown in **c**. The rapid increase of $I_{D(t)}$ of the case colored green caused an increase of the sum of $I_{D(t)}$ and $I_{SH(t)}$. As a result, its $I_{\left( t \right)}$ rapidly decreased as shown in **b**, which formed the “inward curve” phenomenon as shown in **a**.

**Fitting with actual *I-V* curves of PSCs using equation (10)**

**Figure S7** A fitting result with one actual *I-V* curve of PSCs using equation (10). The corresponding PSC was fabricated using the same method described in Figure S1. Three fitting results are presented in the Figure and the values/simulating conditions of the corresponding parameters/variables are listed in the insets with the same color of the relative *I-V* curve. The first fitting process (red colored) was based on the constants listed in the black inset and utilized $nV_{T}$, $I_{0}$, $I_{total}$, $R_{S/OFF}$ and $R_{{SH}/OFF}$ as simulation variables whose computation-step and computation-scope used in the fitting process as well as the computation result are listed in the red inset. By further increasing the computation-scope and decreasing the computation-step of the corresponding simulation variables, a more reasonable fitting result was obtained (blue colored). Based on the two fitting results and the understanding of the effects of the variables on the *I-V* curve, a more satisfactory fitting result was obtained by adjusting certain parameters (purple colored).

The corresponding VBA program is presented in “**VBA Program in Excel for computing the numerical solution of Equation (10)”** above.

1 Liu, M., Johnston, M. B. & Snaith, H. J. Efficient planar heterojunction perovskite solar cells by vapour deposition. *Nature* **501**, 395-398 (2013).
